# Recurrent prostate cancer: combined role for MRI and PSMA-PET in ^68^Ga-PSMA-11 PET/MRI

**DOI:** 10.1007/s00330-023-10442-4

**Published:** 2023-12-01

**Authors:** Kai Jannusch, Nils Martin Bruckmann, Janna Morawitz, Matthias Boschheidgen, Harald H. Quick, Ken Herrmann, Wolfgang P. Fendler, Lale Umutlu, Martin Stuschke, Boris Hadaschik, Gerald Antoch, Lars Schimmöller, Julian Kirchner

**Affiliations:** 1 Department of Diagnostic and Interventional Radiology, Medical Faculty, University Dusseldorf, Moorenstrasse 5, 40225 Dusseldorf, Germany; 2 High-Field and Hybrid MR Imaging, University Hospital Essen, University Duisburg-Essen, 45147 Essen, Germany; 3 Erwin L. Hahn Institute for Magnetic Resonance Imaging, University Duisburg-Essen, 45141 Essen, Germany; 4 Department of Nuclear Medicine, University Hospital Essen, University of Duisburg-Essen, 45147 Essen, Germany; 5 Department of Diagnostic and Interventional Radiology and Neuroradiology, University Hospital Essen, University of Duisburg-Essen, 45147 Essen, Germany; 6 Department of Radiation Oncology, West German Cancer Center, Medical Faculty, University Hospital Essen, Hufelandstr. 55, 45147 Essen, Germany; 7 German Cancer Consortium (DKTK), Partner Site University Hospital Essen, Hufelandstrasse 55, 45147 Essen, Germany; 8 Department of Urology, University of Duisburg-Essen and German Cancer Consortium (DKTK)-University Hospital, Essen, Germany; 9 Center for Integrated Oncology, Aachen Bonn Cologne Düsseldorf (CIO ABCD), Bonn, Germany; 10 Department of Diagnostic, Interventional Radiology and Nuclear Medicine, Marien Hospital Herne, University Hospital of the Ruhr-University Bochum, Herne, Germany

**Keywords:** Positron-emission tomography, Magnetic resonance imaging, Prostate cancer, Tumor staging

## Abstract

**Objectives:**

To investigate the specific strengths of MRI and PET components in ^68^Ga-PSMA-11 PET/MRI for staging of patients with biochemically recurrent prostate cancer (PCa).

**Methods:**

Patients with biochemical recurrence of PCa and contrast-enhanced whole-body ^68^Ga-PSMA-11 PET/MRI including a dedicated pelvic multiparametric MRI were included in this retrospective study. Imaging datasets of MRI and PET were evaluated separately regarding local PCa recurrence (Tr), pelvic lymph node metastases (N1), distant lymph node metastases (M1a), bone metastases (M1b), and soft tissue metastases (M1c) according to PROMISE version 1. Data evaluation was performed patient- and region-/lesion-based. Cox regression revealed a PSA of 1.69 ng/mL as a cut-off for subgroup analysis. Sensitivity, specificity, positive predictive value (PPV), negative predictive value (NPV), and accuracy were evaluated for each image component. Differences in staging accuracy were assessed using the Wilcoxon and McNemar test.

**Results:**

Altogether 102 patients (mean aged 68 ± 8 years, median PSA 1.33 ng/mL) were included. PCa was found in 70/102 (68%) patients. Accuracy of MRI in the detection of Tr, N1, M + , M1a, and M1b was 100%, 79%, 90%, 97%, and 95% for PSA < 1.69 ng/mL and 100%, 87%, 87%, 91%, and 96% for PSA > 1.69 ng/mL. Accuracy of ^68^Ga-PSMA-11 PET was 93%, 97%, 93%, 98%, and 100% for PSA < 1.69 ng/mL and 87%, 91%, 96%, 100%, and 96% for PSA > 1.69 ng/mL.

**Conclusions:**

Combined assessment of ^68^Ga-PSMA-11 PET/MRI improves tumor localization in men with biochemical recurrence. The MRI detected local recurrence of PCa more often whereas ^68^ Ga-PSMA-11 PET detected lymph node metastases more often, especially for PSA < 1.69 ng/mL.

**Clinical relevance statement:**

This study gives a scientific baseline to improve the understanding and reading of ^68^Ga-PSMA-11 PET/MRI imaging in patients with biochemically recurrent PCa by showing the specific strength of each imaging component.

**Key Points:**

*• Combining the individual modality strengths of* ^*68*^ *Ga-PSMA-11 PET/MRI improves tumor localization in men with biochemical recurrence of prostate cancer.*

*• MRI component of* ^*68*^ *Ga-PSMA-11 PET/MRI shows its strength in detecting local recurrence of prostate cancer, especially at PSA < 1.69 ng/mL.*

*•* ^*68*^ *Ga-PSMA-11 PET component shows its strength in detecting local and distant lymph node metastases, especially at PSA < 1.69 ng/mL.*

**Supplementary Information:**

The online version contains supplementary material available at 10.1007/s00330-023-10442-4.

## Introduction

Both ^68^ Ga-PSMA PET and multiparametric MRI play an increasing role in the work-up of prostate cancer (PCa) [1]. After curative therapy with radical prostatectomy (RP) or radiotherapy (RT), recurrence occurs in approximately 20 to 40% of cases, depending on the individual PCa risk category [2, 3]. Follow-up by measuring prostate-specific antigen (PSA) levels is crucial to detect recurrence as soon as possible. At least two PSA levels of 0.2 ng/mL or higher after RP or an increase in PSA of at least 2 ng/mL above the nadir after RT is defined as a biochemical recurrence of PCa [4]. Imaging plays a major role in localizing recurrent disease manifestations and paves the way for further salvage treatment [5, 6]. Therapy options range from curative concepts (e.g., in case of local recurrence) to stereotactic radiation therapy (e.g., in case of distant oligometastasis) and palliation. Therefore, metabolic imaging using prostate-specific membrane antigen (PSMA) PET/CT is taking an increasing role providing both, higher sensitivity and specificity compared to conventional imaging [7–9]. Nonetheless, due to tracer accumulation in the bladder and reduced soft tissue contrast, especially the identification of local recurrence in an early stage can be challenging by Ga-PSMA PET and/or CT while being crucial for therapy decisions [10]. Multiparametric magnetic-resonance-imaging (mpMRI) shows a high soft tissue contrast that might visualize local PCa recurrence more precisely, even in very small lesions [10, 11]. However, MRI is not the modality of choice for detecting small lymph node metastases due to reduced sensitivity and specificity [12, 13]. PET/MRI scanners might be advantageous with a dedicated, body region-focused and multiparametric MRI protocol in combination with a fast whole-body PET protocol (^68^ Ga-PSMA) [14]. Current ^68^ Ga-PSMA PET/MRI studies have primarily taken a competing approach to ^68^ Ga-PSMA PET/CT as well as CT, MRI, or bone scintigraphy, rather than evaluating the complementary information arising from the PSMA-PET and MRI component [15, 16]. Therefore, the aim of this study was to investigate the specific impact of each imaging component at ^68^ Ga-PSMA PET/MRI for staging patients with biochemical PCa recurrence.

## Material and methods

### Patients

The institutional review board (study number 11–4822-BO) approved this study and it was performed in accordance with the Declaration of Helsinki [17]. A general written informed consent form was obtained from all patients undergoing PET/MRI for staging to cover possible further analysis.

This retrospective study included patients with biochemical recurrence of PCa after RP and RT between 01/2015 and 09/2021 at the Department of Nuclear Medicine and Department of Diagnostic and Interventional Radiology (University-Hospital-Essen). All patients underwent a contrast-enhanced whole-body ^68^Ga-PSMA-11 PET/MRI. The minimum requirement for the inclusion of the patients was the presence of at least one T2-weighted (T2w) sequence, one T1w post-contrast sequence, and one PET reconstruction that included the prostate fossa. Furthermore, only patients were included with additional recorded patient characteristics including age-, PSA level at the time of scan, and choice of initial curative treatment. Patients suffering from other second malignancies were excluded from the retrospective data collection. 

### Whole-body/multiparametric pelvic PET/MRI

All ^68^Ga-PSMA-11 PET/MRI examinations were performed on an integrated 3-Tesla PET/MRI system (Biograph mMR, Siemens Healthcare GmbH) with an average delay of 130 ± 61 min after ^68^Ga-PSMA-11 injection. The total mean activity was 111 ± 35 MBq. The field of view of ^68^GaPSMA-11 PET/MRI examinations was chosen from the skull base to the mid-thigh except for 12/102 patients who underwent a single pelvic PET/MRI. ^68^Ga-PSMA-11 PET/MRI was performed using a high-channel surface coil. First, simultaneous PET and 3D-Dixon-VIBE sequences for MRI-based scatter correction were performed (acquisition time 2 min per bed position using static frames). PET was reconstructed using iterative reconstruction (3 iterations, 21 subsets) and a Gaussian filter (4 mm). A standardized whole-body MRI protocol was performed for 90/102 as visualized in Table 1. The following MR sequences were acquired for the whole body protocol: (i) axial 3D Dixon VIBE pre- and post-contrast imaging and (ii) diffusion-weighted sequences (including high b values, *b* = 0, 1000, 1500, 2000), and the following MR sequences were acquired for prostate imaging: (i) high-resolution T2-weighted fast spin-echo (TSE) sequences (axial, coronal, and sagittal planes), (ii) diffusion-weighted sequences (including high b values, *b* = 0, 50, 1000, 1500, 2000), and (iii) dynamic (after RT) or single (after RP) T1w-contrast-enhanced imaging (VIBE sequence). Depending on a steady improvement of the staging algorithm during the long inclusion period and due to occasional premature examination stops, variations in quality and completeness of the acquired mpMRI / whole-body MRI and PET sequences were present.

**Table 1:** Detailed information about the whole-body MRI protocol and the MRI protocol of the prostate fossa separated by sequences and detailed parameters of the sequence

**Sequence (whole body)**	**Parameters**
3D Dixon VIBE	Slice thickness 3.5 mm; TE 1.29 ms; TR 4.05 ms; FOV 380 mm; Voxel size 1.2 × 1.2 × 3.5
Diffusion-weighted sequences (b values = 0, 1000, 1500, 2000)	Slice thickness 5 mm; TE 70 ms; TR 8100 ms; FOV 420 mm; Voxel size 2.6 × 2.6 × 5.0 mm
**Sequence (prostate fossa)**	**Parameters**
T2-weighted TSE sequence	Slice thickness 3.0 mm; TE 101 ms; TR 3740 ms (sagittal), 4360 ms (axial), 4000 ms (coronal); FOV 200 mm; Voxel size 0.6 × 0.6 × 3.0
Diffusion-weighted sequences (b values = 0, 50, 1000, 1500, 2000)	Slice Thickness 3 mm; TE 106 ms; TR 6700 ms; FOV 180 mm; Voxel size 1.6 × 1.6 × 3.0 mm
T1-weighted VIBE sequence	Slice thickness 3.5 mm; TE 1.29 ms; TR 4.05 ms; FOV 380 mm; Voxel size 1.2 × 1.2 × 3.5

### Image analysis

Using the dedicated imaging processing software OsiriX (Pixmeo SARL), imaging datasets of the ^68^Ga-PSMA-11 PET/MRI examination were analyzed by board-certified radiologists and nuclear medicine physicians with experience in reading multiparametric prostate MRI and hybrid imaging and PSMA PET. Readers were aware of the biochemical recurrence of PCa and individual PSA levels but blinded to patient identification data and prior- or follow-up examinations. Blinded to the ^68^Ga-PSMA-11 PET images, MRI whole-body sequences as well as the MRI sequences of the prostate fossa were evaluated by a uro-radiological specialist with more than 12 years of experience. Independently from that and blinded to all MRI sequences, the ^68^Ga-PSMA-11 PET images were evaluated by the hybrid imaging specialist with more than five years of experience. An image quality score (QUAL score) was determined for the MRI component and a 5-point Likert score for the PET component of each ^68^Ga-PSMA-11 PET/MRI examination to address variances in image quality (Table 2) [18]. According to the PI-QUAL score that was developed and well established in treatment-naive patients undergoing prostate cancer detection, a QUAL score was defined for this study in patients with biochemical recurrence of PCa after therapy [18]. The QUAL score was used in the same approach. A QUAL score of five was defined as a fully acquired MRI of the prostate fossa according to the mentioned sequences (see Table 1) with an optimal diagnostic quality. The presence of at least one optimal T1 weighted post-contrast sequence including the prostate fossa was considered as the presence of the contrast-enhanced prostate sequence, even in the absence of dynamic contrast-enhanced imaging. ^68^Ga-PSMA-11 PET image quality was subjectively rated depending on clinical experience according to a well-established 5-point Likert score ranging from one (non-diagnostic) to five (excellent quality) as already used and experienced in several other studies [19, 20].

**Table 2:** Definition of the applied QUAL score (MRI) and 5-point Likert scale (PET) for rating diagnostic / image quality and lesion detectability

Rating	QUAL score for MRI component	5-point Likert scale for PET component
1	All mpMRI sequences are below the minimum standard of diagnostic quality	Non-diagnostic: inability to discern lesions from background
2	Only one mpMRI sequence is of acceptable diagnostic quality	Poor quality: only subtle distinction of lesions from background
3	At least two mpMRI sequences taken together are of diagnostic quality	Moderate quality: ability to discern lesions with significant noise
4	Two or more mpMRI sequences are independent of diagnostic quality	Good quality: ability to discern lesions with low noise
5	All mpMRI sequences are of optimal diagnostic quality	Excellent quality: ability to discern lesions without noise

Localization and conspicuity were evaluated on both, MRI and PET components with respect to (i) local recurrence of PCa (Tr); (ii) pelvic lymph node metastases (N1) and amount (nodal amount; NA); (iii) distant lymph node metastases above the aortic bifurcation (M1a); (iv) bone metastases summing up all categories of bone metastases (M1b); and (v) parenchymal lesions (M1c) according to the PROMISE guidelines (version 1.0) [21]. Readers used a binary nomenclature to distinguish between malignant vs. benign to ensure better comparability. Deviating from the accepted PROMISE classification, the local recurrence of PCa was divided into Tr (local recurrence) and T0 (no local recurrence). Distant metastases (M1a, M1b, M1c) were also concluded to be M + (all distant metastases including bone metastases) [21]. For local- and distant soft-tissue recurrence of PCa, lesions with intermediate intensity on T2w imaging, accentuated contrast enhancement at T1w post-contrast sequence, high signal on diffusion-weighted imaging (DWI), and a low signal on the corresponding apparent diffusion coefficient (ADC) map (indication diffusion disorder) were suspicious for malignancy [15]. Local recurrence of PCa was measured at T1w post-contrast sequence (length x width in mm). In lymph nodes, typical pelvic location, short axis diameter exceeding 10 mm, or short axis diameter between 4 and 10 mm with a spherical configuration in typical localization and/or (suspicious) contrast enhancement were regarded as suspicious for malignancy [22]. Low signal intensity on T1w and high signal intensity on T2w imaging as well as contrast enhancement and diffusion disorder were consistent with bone metastases [15]. For lesion characterization on PET, visually increased focal ^68^Ga-PSMA-11 uptake in comparison to the background tissue was considered indicative of malignancy [23]. SUV_max_ of the index lesion was measured by using a spherical volume of interest.

### Reference standard

For accurate lesion characterization in both modalities, the MRI and ^68^Ga-PSMA-11 PET images were evaluated in a separate consensus reading by both scientifically and clinically highly experienced readers to build an expert reference standard. Changes in previous data evaluation of each reader was not allowed. To improve expert-based reference standard, follow-up- and prior cross-sectional imaging was taken into account. Furthermore, if available clinical information was included from the readers to complete the modified reference standard according to previous publications on PET/MRI [24–26].

### Statistical analysis

SPSS Statistics 26 (IBM Inc.) was used for statistical analysis. PSA-based subgroup analysis after cut-off evaluation using Cox regression (PSA < / > 1.69 ng/mL) was performed. Further subgroup analysis of RP-only patients and non-subgroup comparison was performed as visualized in the supplementary material. Subgroup analysis of RT-only patients was waived due to low, statistically non-suitable sample size. Data analysis was performed patient-based and region-/lesion-based. Descriptive analysis was performed and data are presented as mean ± SD, median, and interquartile range (IQR). Sensitivity, specificity, positive predictive value, negative predictive value, and diagnostic accuracy were calculated for MRI and PET lesion-based evaluation including confidence intervals (CI) at 95%, except for the M1c stage due to the low number of cases. The Wilcoxon test was chosen for the evaluation of differences in tumor stage between MRI and ^68^ Ga-PSMA-11 PET. The McNemar test was used for binary group comparison at region-/lesion-based analysis except for the M1c stage according to the low number of cases. *p* values < 0.05 were considered to be statistically significant.

## Results

In total *n* = 102 patients (mean age: 68 ± 8 years; range 51–83 years) with biochemical recurrence of PCa (median PSA: 1.33 ng/mL) were included in this retrospective study. Radical prostatectomy was performed in 92/102 (90%) patients (median PSA: 1.0 ng/mL) and 10/102 (10%) patients (median PSA: 3.85 ng/mL) received primary radiotherapy. MRI QUAL score revealed a median of 3 (IQR: 1) and PET 5-point Likert scale revealed a median of 4 (IQR: 1). A total of *n* = 38 patients had a MRI QUAL score below 3 and a total of *n* = 2 patients had a PET 5-point Likert scale below 3. According to the cut-off value of PSA 1.69 ng/ml for further subgroup analysis, 58/102 (57%) patients had a PSA value < 1.69 ng/mL, and 44/102 (43%) patients had a PSA value > 1.69 ng/mL.

### Patient-based analysis

Altogether 27/58 (47%) patients (PSA < 1.69 ng/mL) and 43/44 (98%) patients (PSA > 1.69 ng/mL) had a recurrence of PCa according to the reference standard. In the subgroup of PSA < 1.69 ng/mL MRI identified 17/27 (63%) patients correct with five patients rated false positive (5/31, 16%), and ^68^Ga-PSMA-11 PET identified 24/27 (89%) patients correct with two patients rated false positive (2/31, 6%). Subgroup analysis of patients with PSA > 1.69 ng/mL revealed 39/43 (90%) patients correctly identified at MRI with one patient rated false positive and 37/43 (86%) patients correctly identified at ^68^Ga-PSMA-11 PET with one patient rated false positive.

**Fig. 1:**
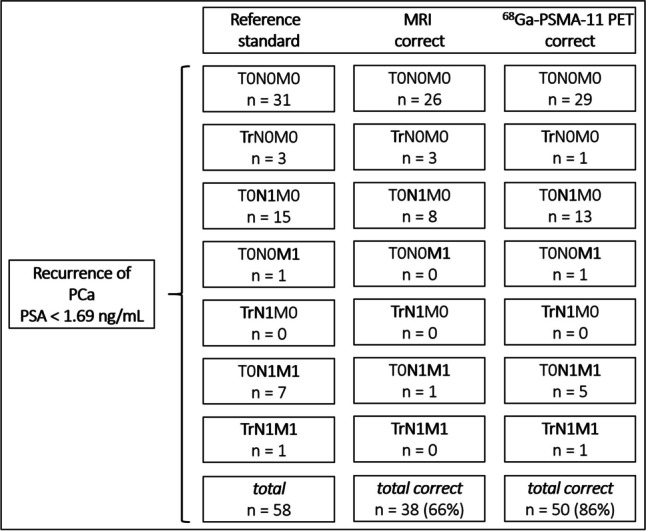
Adapted PROMISE stage of all patients with recurrence of prostate cancer (PCa) according to the reference standard and values of correct assessment by MRI or ^68^Ga-PSMA-11 PET component for PSA < 1.69 ng/mL subgroup

**Fig. 2:**
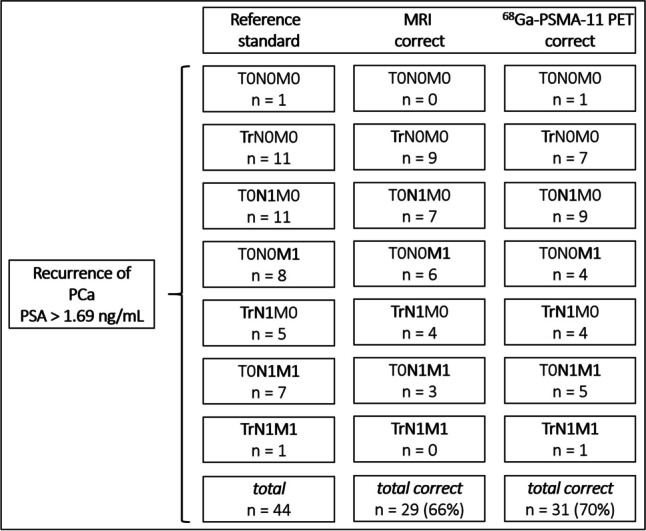
Adapted PROMISE stage of all patients with recurrence of prostate cancer (PCa) according to the reference standard and values of correct assessment by MRI or ^68^Ga-PSMA-11 PET component for PSA > 1.69 ng/mL subgroup

According to the reference standard, all patients were subdivided into seven adapted PROMISE stages as visualized in Fig. 1 for PSA < 1.69 ng/mL and Fig. 2 for PSA > 1.69 ng/mL**.** Due to missing upper abdomen and chest imaging, determining the PROMISE stage was limited in 12 of the total 102 patients of the cohort. Correct tumor stage for PSA < 1.69 ng/mL subgroup was determined by MRI in 38/58 (66%) patients and by ^68^Ga-PSMA-11 PET in 50/58 (86%) patients and for PSA > 1.69 ng/mL subgroup in 29/44 (66%) patients (MRI) and 31/44 (70%) patients (^68^Ga-PSMA-11 PET). There were no significant differences between both imaging components (*p* > 0.05). Further PROMISE stage subgroup analysis of patients after RP and non-subgroup analysis is provided in the supplementary material without significant differences between both imaging components (*p* > 0.05).

### Region- and lesion-based analysis

A statistical overview of PSA subgroup analysis is given in Tables 3 and 4. There were no significant differences in staging between MRI and ^68^Ga-PSMA-11 PET (*p* > 0.05). Further subgroup analysis of patients after RP and non-subgroup analysis also revealed no significant staging differences as visualized in the supplementary material. Sensitivity, specificity, PPV, NPV, and diagnostic accuracy for Tr, N1, M1 + , M1a, and M1b at PSA subgroup analysis are given in Table 5.

**Table 3:** Summary on recurrence of PCa divided into different regions and subdivided by MRI and ^68^Ga-PSMA-11 PET for PSA < 1.69 ng/mL subgroup

Reference standard PSA < 1.69 ng/mL		MRI			^68^Ga-PSMA-11 PET		
		Correct	False positive	False negative	Correct	False positive	False negative
Local recurrence	4/58 (7%)	4/4 (100%)	0	0	2/4 (50%)	2/54 (4%)	2/4 (50%)
Pelvic LNM	23/58 (40%)	13/23 (57%)	3/35 (9%)	10/23 (43%)	22/23 (96%)	1/35 (3%)	1/23 (4%)
M +	9/58 (16%)	4/9 (44%)	1/49 (2%)	5/9 (56%)	5/9 (56%)	0	4/9 (44%)
M1a	2/58 (3%)	0	0	2/2 (100%)	1/2 (50%)	0	1/2 (50%)
M1b	6/58 (10%)	4/6 (67%)	1/52 (2%)	2/6 (33%)	3/6 (50%)	0	3/6 (50%)
M1c	1/58 (2%)	0	0	1/1 (100%)	1/1 (100%)	0	0

**Table 4:** Summary on recurrence of PCa divided into different regions and subdivided by MRI and ^68^Ga-PSMA-11 PET for PSA > 1.69 ng/mL subgroup

Reference standard PSA > 1.69 ng/mL		MRI			^68^Ga-PSMA-11 PET		
		Correct	False positive	False negative	Correct	False positive	False negative
Local recurrence	17/44 (39%)	8/8 (100%)	0	0	12/17 (71%)	1/27 (4%)	5/17 (29%)
Pelvic LNM	24/44 (55%)	20/44 (45%)	2/20 (10%)	4/44 (9%)	22/44 (50%)	2/20 (10%)	2/44 (5%)
M +	19/44 (43%)	15/19 (79%)	2/25 (8%)	4/19 (21%)	17/19 (89%)	0	2/19 (11%)
M1a	9/44 (20%)	5/9 (56%)	0	4/9 (44%)	9/9 (100%)	0	0
M1b	9/44 (20%)	9/9 (100%)	2/35 (6%)	0	7/9 (78%)	0	2/9 (22%)
M1c	1/44 (2%)	1/1 (100%)	0	0	1/1 (100%)	0	0

**Table 5:** Subgroup analysis of patients with PSA above and below 1.69 ng/mL. Sensitivity, specificity, positive predictive value, negative predictive value, and diagnostic accuracy for each modality (MRI vs. ^68^Ga-PSMA-11 PET) subdivided into local recurrence (Tr), pelvic lymph node metastases (N1), combined distant recurrence (M +), distant lymph node metastases (M1a) and bone metastases (M1b). The 95% confidence interval (CI) is given for each value. Bold indicates missing overlap of CI

PSA < 1.69 ng/mL	MRI					^68^Ga-PSMA-11 PET				
	Tr	N1	M +	M1a	M1b	Tr	N1	M +	M1a	M1b
Sensitivity	100 CI (95%): 40–100	57 CI (95%): 34–77	44 CI (95%): 14–79	0 CI (95%): 0–84	67 CI (95%): 22–96	50 CI (95%): 7–93	96 CI (95%): 78–100	56 CI (95%): 21–86	50 CI (95%): 1–99	100 CI (95%): 3–100
Specificity	100 CI (95%): 93–100	92 CI (95%): 79–98	98 CI (95%): 89–100	100 CI (95%): 94–100	98 CI (95%): 90–100	96 CI (95%): 88–100	97 CI (95%): 85–100	100 CI (95%): 93–100	100 CI (95%): 94–100	100 CI (95%): 94–100
Positive predictive value	100 CI (95%): 40–100	81 CI (95%): 54–96	80 CI (95%): 28–99	– CI (95%): 0–100	80 CI (95%): 28–99	50 CI (95%): 7–93	96 CI (95%): 78–100	100 CI (95%): 48–100	100 CI (95%): 3–100	100 CI (95%): 3–100
Negative predictive value	100 CI (95%): 93–100	78 CI (95%): 63–89	91 CI (95%): 80–97	97 CI (95%): 88–100	96 CI (95%): 87–100	96 CI (95%): 88–100	97 CI (95%): 85–100	93 CI (95%): 82–98	98 CI (95%): 91–100	100 CI (95%): 94–99
Accuracy	100 CI (95%): 94–100	79 CI (95%): 66–88	90 CI (95%): 79–96	97 CI (95%): 88–100	95 CI (95%): 86–99	93 CI (95%): 84–98	97 CI (95%): 88–99	93 CI (95%): 83–98	98 CI (95%): 91–100	100 CI (95%): 94–100
**PSA > 1.69 ng/mL**										
Sensitivity	100 CI (95%): 63–100	83 CI (95%): 63–95	79 CI (95%): 54–94	56 CI (95%): 21–86	100 CI (95%): 67–100	71 CI (95%): 44–90	92 CI (95%): 73–99	90 CI (95%): 67–99	100 CI (95%): 66–100	78 CI (95%): 40–97
Specificity	100 CI (95%): 87–100	91 CI (95%): 71–99	93 CI (95%): 76–99	100 CI (95%): 90–100	95 CI (95%): 82–99	96 CI (95%): 82–100	91 CI (95%): 71–99	100 CI (95%): 86–100	100 CI (95%): 90–100	100 CI (95%): 90–100
Positive predictive value	100 CI (95%): 63–100	91 CI (95%): 71–99	88 CI (95%): 64–99	100 CI (95%): 48–100	82 CI (95%): 48–98	92 CI (95%): 30–93	92 CI (95%): 73–99	100 CI (95%): 67–100	100 CI (95%): 66–100	100 CI (95%): 59–100
Negative predictive value	100 CI (95%): 87–100	83 CI (95%): 63–95	86 CI (95%): 68–96	90 CI (95%): 76–97	100 CI (95%): 90–100	84 CI (95%): 67–95	91 CI (95%): 71–99	93 CI (95%): 76–99	100 CI (95%): 90–100	95 CI (95%): 82–9
Accuracy	100 CI (95%): 90–100	87 CI (95%): 74–95	87 CI (95%): 74–95	91 CI (95%): 78–97	96 CI (95%): 88–98	87 CI (95%): 85–99	91 CI (95%): 79–98	96 CI (95%): 85–99	100 CI (95%): 92–100	96 CI (95%): 85–99

#### Local recurrence (Tr stage)

**Fig. 3:**
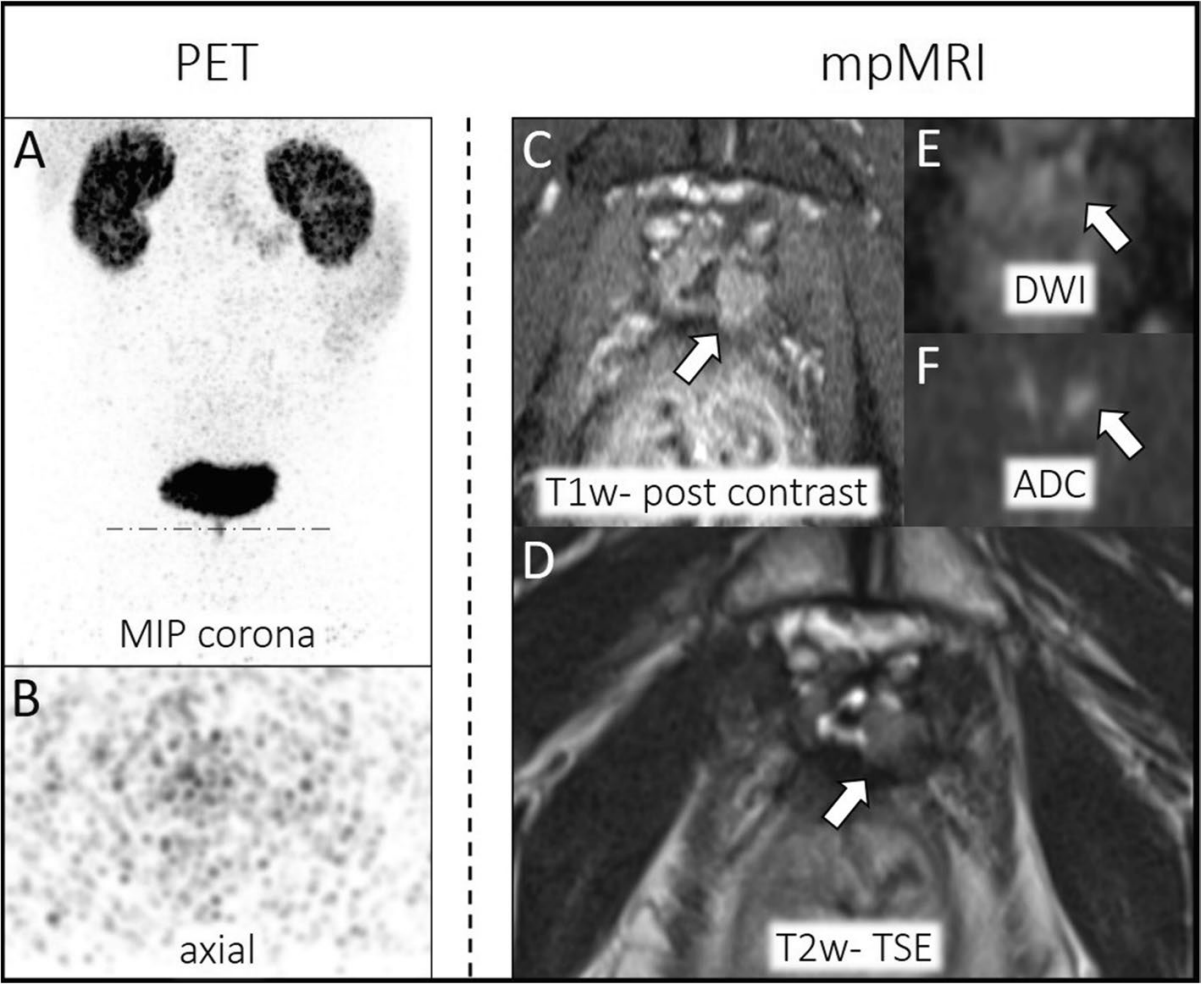
Local recurrence of PCa arising from the vesico-urinary anastomosis. No suspicious uptake is visible in the ^68^Ga-PSMA-11 PET component (left **A**-**B**: MIP corona, axial dataset) due to tracer accumulation in the bladder. Dashed line in MIP corona (**A**) indicates axial PET layer (**B**). MpMRI depicts tumor relapse (right **C**-**F**, white arrow: T1w post contrast, T2w-TSE; DWI, ADC)

In PSA < 1.69 ng/mL subgroup 4/58 (7%) patients and in PSA > 1.69 ng/mL subgroup 17/44 (39%) patients had local recurrence according to the reference standard. MRI identified all Tr stages (see Tables 3 and 4). The mean size of local recurrence was 14 ± 4 (PSA < 1.69 ng/mL) and 19 ± 8 (PSA > 1.69 ng/mL). Missed local recurrence at ^68^Ga-PSMA-11 PET evaluation revealed a maximal diameter of 10 ± 1 mm (PSA < 1.69 ng/mL) and 21 ± 9 (PSA > 1.69 ng/mL). An example is visualized in Fig. 3.

#### Pelvic lymph node metastases (N stage)

**Fig. 4:**
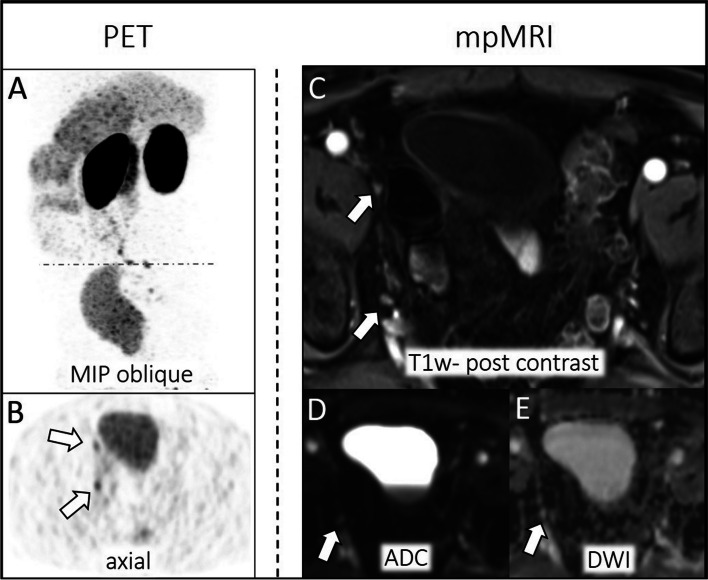
Pelvic lymph node recurrence of PCa parailiacal right. Lymph node metastases are detected by tracer accumulation (SUVmax: 9.3) at ^68^Ga-PSMA-11 PET (left **A**-**B**, white arrow: MIP oblique, axial dataset). Dashed line in MIP corona (**A**) indicates axial PET layer (**B**). Unsuspicious lymph node appearance at mpMRI (right **C**-**E**, white arrow: T1w post contrast, ADC, DWI)

In the PSA < 1.69 ng/mL subgroup and the PSA > 1.69 ng/mL subgroup, 23/58 (40%) and 24/44 (55%) patients, respectively, had pelvic lymph node recurrence according to the reference standard. There was a total of 50 lymph node metastases in patients with PSA < 1.69 ng/mL and a total of 36 lymph node metastases in patients with PSA > 1.69 ng/mL. In MRI 20/50 (40%) lymph node metastases were identified by MRI whereas three were rated false positive for PSA < 1.69 ng/mL and 30/50 (60%) lymph node metastases for PSA > 1.69 ng/mL whereas two were rated false positive. Missed lymph node metastases at MRI evaluation revealed a maximal diameter of 3 mm ± 1 mm (PSA < 1.69 ng/mL) and 5 mm ± 2 mm (PSA > 1.69 ng/mL). Based on the ^68^Ga-PSMA-11 PET 34/50 (68%) lymph node metastases were identified for PSA < 1.69 ng/mL whereas one was rated false positive and 41/50 (82%) lymph node metastases were identified for PSA > 1.69 ng/mL whereas two were rated false positive. For an example, see Fig. 4. There is no CI overlap between MRI and ^68^Ga-PSMA-11 PET sensitivity in the PSA < 1.69 ng/mL subgroup (see Table 5).

**Fig. 5:**
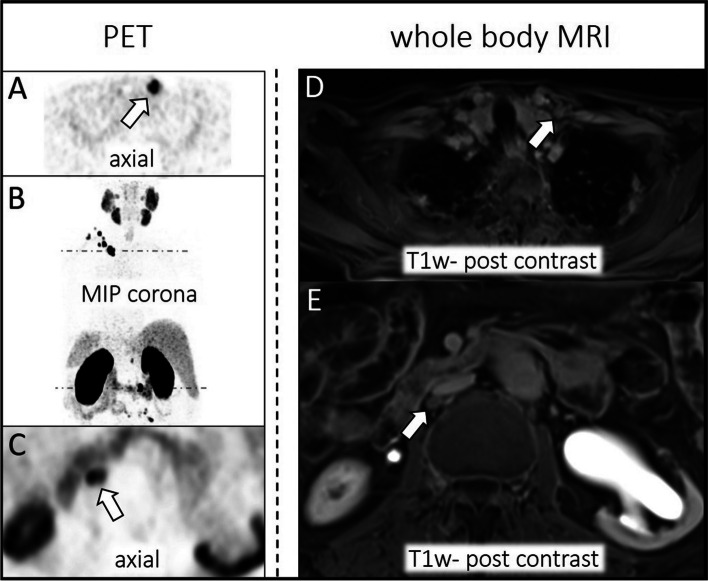
Distant lymph node recurrence of PCa. Cervical (**A**) and retroperitoneal (**C**) lymph node metastases detectable by strong tracer accumulation (SUVmax: 60.3) at ^68^Ga-PSMA-11 PET (left **A**-**C**, white arrow: MIP corona, axial datasets). Dashed line in MIP corona (**B**) indicates axial PET layer (**A**, **B**). No pathological rating cervical (**D**) and retroperitoneal (**E**) at mpMRI (right, white arrow: T1w post contrast)

**Fig. 6:**
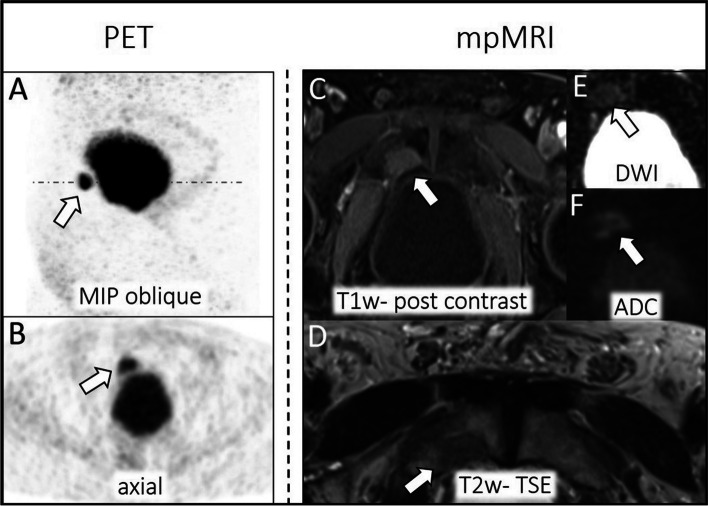
Bone metastases of PCa at os pubis right. Tracer accumulation (SUVmax: 10.9) was misinterpreted as lymph node metastases at ^68^Ga-PSMA-11 PET (left **A**-**B**, white arrow: MIP oblique, axial dataset). Dashed line in MIP oblique (**A**) indicates axial PET layer (**B**). Correct rating at mpMRI (right **C**-**F**, white arrow: T1w post contrast, T2w- TSE, DWI, ADC)

Detailed results of N stage subgroup analysis as well as further analysis of the M1 + stage, M1a stage, the M1b stage, and M1c stage are given in Tables 3 and 4 with no significant differences in staging between both imaging components (*p* > 0.05). A patient with the M1a stage is exemplified in Fig. 5 and a patient with the M1b stage is exemplified in Fig. 6.

## Discussion

According to both subgroups but especially for patients with lower PSA values, patient-based evaluation showed a general superiority of ^68^Ga-PSMA-11 PET component in identifying the localization of PCa recurrence. Nonetheless, the MRI component was superior in the detection of local recurrence (Tr stage). This trend was also existent in RP subgroup analysis and non-subgroup analysis. There were no significant statistical differences between both imaging components in patient-based evaluation. Region- and lesion-based analysis also revealed no statistically significant differences between both imaging components in detecting Tr, N1, M1 + , M1a, M1b, and M1c stage. Especially for patients with PSA values < 1.69 ng/mL the ^68^Ga-PSMA-11 PET component detected N1 stage more reliably (no CI overlap) than the MRI component.

MpMRI was highly sensitive in detecting local PCa recurrence and detected all local tumor relapses in both subgroups. This underlines the current literature highlighting the high value of MRI in detecting local recurrence based on its very good spatial resolution, soft tissue contrast, and the possibility of a dedicated dynamic contrast-enhanced (DCE) sequence [10, 11, 27–30]. Detection of a small local PCa recurrence can be challenging in ^68^Ga-PSMA-11 PET, especially as curative prostatectomy is usually associated with postoperative bladder descent into the prostatic fossa and most recurrences of PCa are localized at the vesico-urethral anastomosis. This region is often masked in PET images by tracer accumulation into the bladder (hot bladder), especially by using a ^68^ Ga-PSMA tracer [10, 15]. Therefore, local tumor relapse was missed in 50% (PSA < 1.69 ng/mL) and 29% (PSA > 1.69 ng/mL) of the patients in our study. The use of ^18^F-PSMA tracers might solve this problem due to its non-urinary excretion as highlighted by Giesel and colleagues but is also accompanied by a larger amount of false positive bone lesions and higher inter-reader variability [31].

Publications highlighted repeatedly the benefit of ^68^Ga-PSMA PET in the detection of pelvic lymph node metastases compared to conventional imaging like CT or MRI [11, 29]. This is in line with the results of this study, and especially for patients with PSA values below 1.69 ng/mL reporting accuracy of 79% for mpMRI and 97% for ^68^Ga-PSMA-11 PET. Although there were no significant differences in staging between these two imaging components at group comparison, the borderline overlap of the CI for accuracy and no overlap for sensitivity indicates a clinically relevant difference in staging potential that might be not significant due to the limited number of patients. The superior detection rate is explainable by the high prevalence of pelvic lymph node metastases not reaching a pathologic threshold of 10 mm diameter (short-axis), especially in lower PSA values [22, 32, 33]. At our data evaluation, missed lymph node metastases at MRI revealed a maximum diameter (short-axis) of 3–5 ± 1–2 mm according to the PSA subgroup. Even though Valentin et al (2022) promote that in typical localizations and with a round-shaped appearance metastasis up to 4 mm could be detected [22], lymph node metastases with a short axis diameter below 10 mm are challenging to identify at mpMRI. Especially in these very small and therefore early lymph node metastases, the accumulation of prostate-specific tracer enables their detection.

Detection of distant metastases showed non-significant but better performance of the ^68^ Ga-PSMA-11 PET component. However, similar to the localization of pelvic recurrences, the strength of each available imaging component was further evaluated separately for lymph nodes and bone metastases. Consistent with the detection of pelvic lymph node metastases, the ^68^ Ga-PSMA-11 PET slightly outperformed MRI component in detecting distant lymph node metastases (M1a) in this study for both subgroups, reporting accuracy of 97% and 91% for mpMRI (PSA < / > 1.69 ng/mL) as well as 98% and 100% for ^68^ Ga-PSMA-11 PET (PSA < / > 1.69 ng/mL). Some studies revealed that early stages of bone metastases can be detected more easily at MRI by changes in signal intensities at MRI due to growing malignant cell clusters in bone marrow [34, 35]. However, there are prospective data that prove the superiority of PSMA PET over MRI for M1b staging, taking into account that only diffusion-weighted imaging of MRI was used for comparison [36]. While the superiority of MRI could be visualized for patients with PSA values > 1.69 ng/mL, the reduced sensitivity of the ^68^Ga-PSMA-11 PET component visualized in the PSA subgroup with PSA values < 1.69 ng/mL could be caused by misinterpretation of small bone lesions due to the missing anatomical information by evaluating single PET datasets. Thus, bone metastases might be misinterpreted as lymph node metastases due to their close anatomical location to the pelvic bone. The higher MRI false-positive rate in our study makes it difficult to assume a clear superiority of MRI regarding bone metastases. It should be mentioned, that the reference standard is only based on PET/MRI examinations that were evaluated on an expert level in consensus and potential existing cross-sectional imaging. Thus, due to missing long time follow-up, the reference standard has its weakness, especially with a focus on bone metastases and the underestimation of false-positive bone findings at MRI might be possible.

Based on these data, our study comprises two main messages that we believe to be important: First, ^68^ Ga-PSMA-11 PET/MRI is an excellent synergistic imaging modality in the setting of biochemical PCa recurrence, as both imaging components have their specific strengths. Second, as running a PET/MR system still comes at markedly higher costs compared to PET/CT and has limited availabilities, additional mpMRI of the pelvis is highly important after therapy, especially in cases where PET/CT showed no lesion of local recurrence. Generally, a combination of both modalities (PET/CT and mpMRI) seems to be favorable for patient management and is more easily assessable in clinical routine [37, 38]. This is also highlighted in the study of Sonni et al (2021), which presented a comparable performance of combined PET/CT and mpMRI to ^68^ Ga-PSMA-11 PET/MRI in the detection and intraprostatic localization of prostate cancer [38]. Especially in PSMA negativity of prostate cancer (approximately 10%), the multiparametric dataset and high soft tissue contrast of MRI are beneficial for detecting the recurrence of prostate cancer [39, 40]. In general, we would like to emphasize here that a high level of expertise in the evaluation of mpMRI of the prostate is key to its valuable use [41].

This study has some limitations. First, due to evolving image quality in mpMRI in the last decade, some of the included datasets have limited image quality (QUAL 3), bearing the problem that especially the ability to detect pelvic lymph node metastases by MRI might be underestimated. Furthermore, patients with RP did not undergo a contrast dynamic of the prostate fossa in the period of data acquisition, which was considered in the given QUAL score. Second, due to the retrospective design, we only had information about recurrence by PSA levels and no information about the risk category of initial PCa. Thus, a group comparison of risk categories was not possible at all. Third, the reference standard is limited due to missing long time follow-up. Due to the reduced number of patients included in this study, we describe non-significant results according to our data evaluation. Nevertheless, we can visualize trends according to the different subgroups. It should be mentioned, that this kind of PET/MRI data of patients with prostate cancer recurrence are rare. Further larger and possible multicenter studies are needed which might bring our results to a significant level.

Concluding, the MRI component shows its strength in detecting local recurrence of PCa whereas ^68^ Ga-PSMA-11 PET component shows its strength in detecting lymph node metastases, especially for patients with PSA < 1.69 ng/mL without significant statistical staging differences. Nonetheless, the results support the combination of both imaging components for disease localization at PCa recurrence.

### Supplementary Information

Below is the link to the electronic supplementary material.

Supplementary file1 (PDF 360 KB)

## Abbreviations

^68^ Ga: ^68^Gallium
ADC: Apparent diffusion coefficient
DWI: Diffusion-weighted imaging
mpMRI: Multiparametric magnetic-resonance-imaging
NPV: Negative predictive value
PCa: Prostate cancer
PET/MRI: Positron-emission tomography/magnetic resonance imaging
PPV: Positive predictive value
PROMISE: Prostate cancer molecular imaging standardized evaluation
PSA: Prostate-specific antigen
PSMA: Prostate-specific membrane antigen
QUAL: MRI imaging quality score
RP: Radical prostatectomy
RT: Radiotherapy

## Declarations

### Guarantor

The scientific guarantor of this publication is Kai Jannusch.

### Statistics and biometry

One of the authors has significant statistical expertise.

### Study subjects or cohorts overlap

No study subjects or cohorts have been previously reported.

### Methodology

• Retrospective

• Diagnostic or prognostic study

• Performed at one institution
