# Supplementary material for: Recurrent prostate cancer: combined role for MRI and PSMA-PET in 68Ga-PSMA-11 PET/MRI
Source: Eur Radiol. 2023 Dec 1;34(7):4789–800. doi: 10.1007/s00330-023-10442-4 (PMC11213774; doi:10.1007/s00330-023-10442-4)
Supplement: Supplementary file 1 — Supplementary file1 (PDF 360 KB) [file 330_2023_10442_MOESM1_ESM.pdf]

Recurrent prostate cancer: Combined role for MRI and PSMA-PET in 68Ga-PSMA-11 PET/MRI

Electronic Supplementary Material

Patient-based analysis

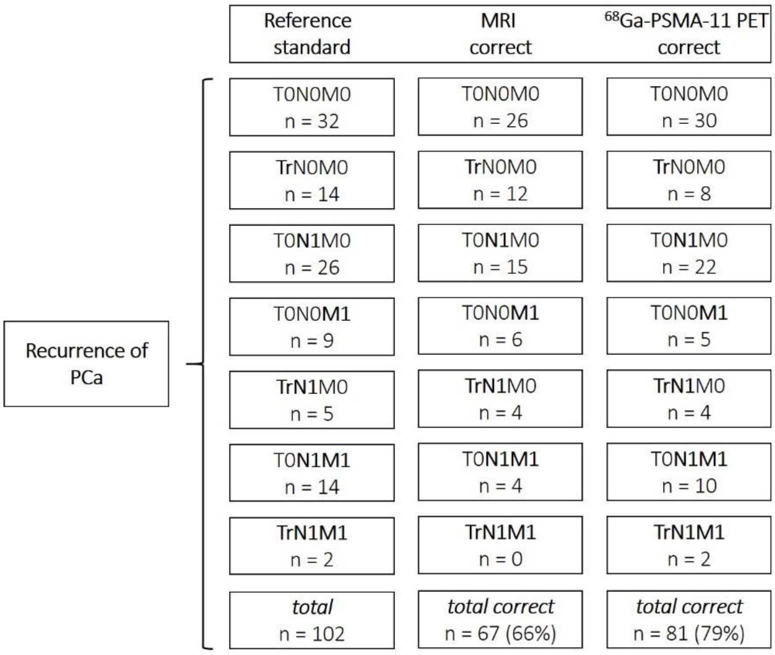

Supplemental Figure 1 Combined non-Subgroup analysis of patients after radical prostatectomy (RP) and radiotherapy (RT) without significant group differences ($p > 0.05$) between both modalities. Adapted PROMISE stage of all patients with recurrence of prostate cancer (PCa) according to the reference standard and values of correct assessment by MRI or ^{68}Ga-PSMA-11 PET component.

Recurrence of Pca after RP

Reference standard	MRI correct	68Ga-PSMA-11 PET correct
T0N0M0 n = 31	T0N0M0 n = 25	T0N0M0 n = 29
TrN0M0 n = 9	TrN0M0 n = 9	TrN0M0 n = 3
T0N1M0 n = 26	T0N1M0 n = 15	T0N1M0 n = 22
T0N0M1 n = 8	T0N0M1 n = 5	T0N0M1 n = 5
TrN1M0 n = 3	TrN1M0 n = 2	TrN1M0 n = 2
T0N1M1 n = 14	T0N1M1 n = 4	T0N1M1 n = 10
TrN1M1 n = 1	TrN1M1 n = 0	TrN1M1 n = 1
total n = 92	*total correct* n = 60 (65%)	*total correct* n = 72 (78%)

Supplemental Figure 2 Subgroup analysis of patients after radical prostatectomy (RP) without significant group differences (p > 0.05) between both modalities. Adapted PROMISE stage of all patients with recurrence of prostate cancer (PCa) according to the reference standard and values of correct assessment by MRI or ^{68}Ga-PSMA-11 PET component.

Region- and lesion-based analysis

Supplemental Table 1 Combined non-Subgroup analysis of patients after radical prostatectomy (RP) and radiotherapy (RT) without significant group differences (*p* > 0.05) between both modalities. Summary on recurrence of PCa divided into different regions and subdivided by MRI and ^{68}Ga-PSMA-11 PET.

Reference standard			MRI				^{68}Ga-PSMA-11 PET		
			correct	false positive	false negative		correct	false positive	false negative
local recurrence	21/102 (21%)		21/21 (100%)	1/81 (1%)	0		14/21 (67%)	3/81 (4%)	7/21 (33%)
pelvic LNM	47/102 (21%)		33/47 (70%)	5/55 (9%)	14/47 (30%)		44/47 (94%)	3/55 (6%)	3/47 (6%)
M+	28/102 (21%)		19/28 (68 %)	3/74 (4%)	9/28 (32%)		22/28 (79%)	0/74	6/28 (21%)
M1a	11/102 (11%)		5/11 (45%)	0	6/11 (54%)		10/11 (91%)	0	1/11 (9%)
M1b	15/102 (21%)		13/15 (87%)	3/87 (3%)	2/15 (13%)		10/15 (67%)	0	5/15 (33%)
M1c	2/102 (2%)		1/2 (50%)	0	1/2 (50%)		2/2 (100%)	0	0

Supplemental Table 2 Combined non-Subgroup analysis of patients after radical prostatectomy (RP) and radiotherapy (RT). Sensitivity, specificity, positive predictive value, negative predictive value and diagnostic accuracy for each modality (MRI vs. ^{68}Ga-PSMA-11 PET) subdivided in local recurrence (Tr), pelvic lymph node metastases (N1), combined distant recurrence (M+), distant lymph node metastases (M1a) and bone metastases (M1b). The 95% confidence interval (CI) is given for each value.

	MRI					^{68}Ga-PSMA-11 PET				
	Tr	N1	M+	M1a	M1b	Tr	N1	M+	M1a	M1b
Sensitivity	100 CI (95%): 84-100	70 CI (95%): 55-83	68 CI (95%): 48-84	45 CI (95%): 17-78	87 CI (95%): 60-98	67 CI (95%): 43-85	94 CI (95%): 82-99	79 CI (95%): 59-92	91 CI (95%): 59-100	67 CI (95%): 38-88
specificity	98 CI (95%): 93-100	91 CI (95%): 80-97	96 CI (95%): 87-99	100 CI (95%): 96-100	97 CI (95%): 90-99	96 CI (95%): 90-99	95 CI (95%): 85-99	100 CI (95%): 95-100	100 CI (95%): 96-100	100 CI (95%): 86-100
positive predictive value	95 CI (95%): 77-100	87 CI (95%): 72-96	86 CI (95%): 65-97	100 CI (95%): 48-100	81 CI (95%): 54-96	82 CI (95%): 57-96	94 CI (95%): 82-99	100 CI (95%): 85-100	100 CI (95%): 69-100	100 CI (95%): 69-100
negative predictive value	100 CI (95%): 95-100	78 CI (95%): 66-87	89 CI (95%): 80-95	94 CI (95%): 87-98	98 CI (95%): 92-100	92 CI (95%): 84-97	95 CI (95%): 85-99	93 CI (95%): 84-97	99 CI (95%): 94-99	94 CI (95%): 88-98
accuracy	99 CI (95%): 95-100	81 CI (95%): 72-88	88 CI (95%): 80-94	94 CI (95%): 88-98	95 CI (95%): 89-98	90 CI (95%): 83-95	94 CI (95%): 88-98	94 CI (95%): 88-98	99 CI (95%): 95-100	95 CI (95%): 89-98

Supplemental Table 3 Subgroup analysis of patients after radical prostatectomy (RP) without significant group differences (p > 0.05) between both modalities. Summary on recurrence of PCa divided into different regions and subdivided by MRI and ^{68}Ga-PSMA-11 PET.

Reference standard RP patients			MRI				^{68}Ga-PSMA-11 PET		
			correct	false positive	false negative		correct	false positive	false negative
local recurrence	13/92 (3%)		13/13 (100%)	1/81 (1%)	0		6/13 (46%)	3/79 (4%)	7/13 (54%)
pelvic LNM	44/92 (48%)		31/44 (70%)	4/48 (8%)	13/44 (30%)		41/44 (93%)	3/48 (6%)	3/44 (7%)
M+	26/92 (28%)		18/26 (69%)	3/66 (5%)	8/26 (31%)		21/26 (80%)	0/66	5/26 (19%)
M1a	10/92 (11%)		5/10 (50%)	0	5/10 (50%)		9/10 (90%)	0	1/10 (10%)
M1b	14/92 (15%)		12/14 (86%)	3/78 (4%)	2/14 (14%)		10/14 (71%)	0	4/14 (29%)
M1c	2/92 (2%)		1/2 (50%)	0	1/2 (50%)		2/2 (100%)	0	0

Supplemental Table 4 Subgroup analysis of patients after radical prostatectomy (RP). Sensitivity, specificity, positive predictive value, negative predictive value and diagnostic accuracy for each modality (MRI vs. ^{68}Ga-PSMA-11 PET) subdivided in local recurrence (Tr), pelvic lymph node metastases (N1), combined distant recurrence (M+), distant lymph node metastases (M1a) and bone metastases (M1b). The 95% confidence interval (CI) is given for each value.

RP patients	MRI					^{68}Ga-PSMA-11 PET				
	Tr	N1	M+	M1a	M1b	Tr	N1	M+	M1a	M1b
Sensitivity	100 CI (95%): 75-100	71 CI (95%): 55-83	69 CI (95%): 48-86	50 CI (95%): 19-81	86 CI (95%): 57-98	46 CI (95%): 19-75	93 CI (95%): 81-99	81 CI (95%): 60-93	90 CI (95%): 55-100	71 CI (95%): 42-92
specificity	99 CI (95%): 93-100	92 CI (95%): 80-98	96 CI (95%): 87-99	100 CI (95%): 96-100	96 CI (95%): 90-99	96 CI (95%): 89-99	94 CI (95%): 82-99	100 CI (95%): 95-100	100 CI (95%): 96-100	100 CI (95%): 95-100
positive predictive value	93 CI (95%): 66-100	89 CI (95%): 76-98	86 CI (95%): 64-97	100 CI (95%): 48-100	80 CI (95%): 52-96	67 CI (95%): 30-93	93 CI (95%): 81-99	100 CI (95%): 84-100	100 CI (95%): 66-100	100 CI (95%): 69-100
negative predictive value	100 CI (95%): 95-100	77 CI (95%): 64-87	89 CI (95%): 79-95	94 CI (95%): 87-98	97 CI (95%): 90-100	92 CI (95%): 83-96	94 CI (95%): 82-99	93 CI (95%): 84-98	99 CI (95%): 93-100	95 CI (95%): 88-99
accuracy	99 CI (95%): 94-100	82 CI (95%): 72-89	88 CI (95%): 80-94	95 CI (95%): 88-98	95 CI (95%): 88-98	89 CI (95%): 81-95	94 CI (95%): 86-98	95 CI (95%): 88-98	99 CI (95%): 96-100	96 CI (95%): 89-99
